# Follitropin Delta as a State-of-the-Art Incorporated Companion for Assisted Reproductive Procedures: A Two Year Observational Study

**DOI:** 10.3390/medicina57040379

**Published:** 2021-04-14

**Authors:** Bogdan Doroftei, Ovidiu-Dumitru Ilie, Ana-Maria Dabuleanu, Roxana Diaconu, Radu Maftei, Gabriela Simionescu, Ciprian Ilea

**Affiliations:** 1 Faculty of Medicine, University of Medicine and Pharmacy “Grigore T. Popa”, University Street, no 16, 700115 Iasi, Romania; bogdandoroftei@gmail.com (B.D.); dabu_93@yahoo.com (A.-M.D.); dr.radu.maftei@gmail.com (R.M.); gabi.ginecologie@gmail.com (G.S.); cilea1979@yahoo.com (C.I.); 2 Clinical Hospital of Obstetrics and Gynecology “Cuza Voda”, Cuza Voda Street, no 34, 700038 Iasi, Romania; dr.diaconuroxana@gmail.com; 3 Origyn Fertility Center, Palace Street, no 3C, 700032 Iasi, Romania; 4 Department of Biology, Faculty of Biology, "Alexandru Ioan Cuza" University, Carol I Avenue, no 20A, 700505 Iasi, Romania

**Keywords:** follitropin delta, ovarian stimulation, pregnancy, reliability, viability

## Abstract

*Background and objectives*: The latest reports suggest that follitropin delta is a highly efficient recombinant human follicle-stimulating hormone (r-hFSH) that became a part of the current assisted reproductive technologies (ARTs). Therefore, the present study aims to assess a series of parameters (follicles, oocytes, and embryos) and further by the outcomes in women following the administration of follitropin delta. *Materials and methods*: This observational study included 205 women. They were aged between 21 and 43 years (mean 33.45) and an anti-Müllerian hormone (AMH) level ranging from 0.11 to 16.00 ng/dL (mean 2.89). *Results*: In accordance with the established methodology and following the centralization of data, a total of fifty-eight pregnancies (28.29%) were achieved; forty-five (36.88%) were achieved in women under 35 years and thirteen (15.66%) in women above 35 years. These figures are positively correlated with women’s age considering that the number of follicles >18 mm, oocytes fertilized and embryo(s) varies among groups. Regarding the interest parameters, we noted *n* = 1719 follicles > 18 mm, *n* = 1279 retrieved oocytes, and *n* = 677 embryos at day 3. On the other hand, the following figures have been registered in women above 35 years: 814–follicles > 18 mm, 612 oocytes retrieved and 301 embryos at day 3. During this study, we registered only three cases of abortions (*n* = 1–0.81% in women under 35 years and *n* = 2–2.40% in women above 35 years). Nine pregnancies (7.37%) were stopped from evolution in females under 35 years, and twelve pregnancies (*n* = 8–6.55% in women under 35 years, while *n* = 4 in women above 35 years) were unsuccessful. A twin pregnancy has been confirmed (1.20%) in women above 35 years, six ongoing pregnancies (4.91%) in those under 35 years, and two in both groups (one per group–*n* = 1–0.81%, and 1.20%–*n =* 1) in which we did not know the exact result were registered at the end of the established studied interval. However, there were also situations in which the treatment cause an over-reactivity or had no effect; *n =* 2 were non-responders, and *n =* 1 exhibited moderate ovarian hyperstimulation syndrome (OHSS). *Conclusions*: Based on our results, we strongly encourage the use of this recombinant gonadotropin on a much larger scale.

## 1. Introduction

Infertility is an intriguing and highly debated topic due to a lack of viable treatment. Fortunately, the fulminant ascension of recombinant DNA technologies promoted the current success of in vitro fertilization (IVF) procedures. Therefore, scientists were able to optimize these protocols and to create recombinant gonadotropins. Thus, human follicle-stimulating hormones (r-hFSHs) became the method of choice in current approaches [1].

Three r-hFSHs are administered within the current clinical practice as part of the assisted reproductive technology (ART) protocols [2]. The fourth is still under development. Follitropin delta is the only human-derived r-hFSH that possesses a defined dosing algorithm [3]. Considering the half-life of endogenous follicle-stimulating hormone (FSH), a long-acting formula was necessary.

Compared with follitropin alfa and/or beta [4,5] and corifollitropin alfa [6], whose pharmacokinetics is calculated based on a 150 International Unit (UI) dose, follitropin deltas emphasize the actual tendency. More precisely it ensures the transition between standardized and personalized doses [7]. Specifically, corifollitropin alfa is a molecular hybrid, with it an effect that ranges between 59 and 82 h. Its terminal rate varies from 68.2 to 73.1 h at 100 to 150 µg at 0.225 L/h. However, it is dependent on the body mass index (BMI) [6].

The anti-Müllerian hormone (AMH) level is the main predictor to set out ovarian reserve and the response to exogenous gonadotropins. The body mass index (BMI), is used, on the other hand, as a determinant after exposure to follitropin delta [8,9,10,11].

The present study aimed to assess a series of parameters: (I) number of follicles, (II) of oocytes, (III) embryo(s) transferred, and (IV) outcomes over a period of twenty-four months among infertile women following the administration of follitropin delta.

## 2. Materials and Methods

### 2.1. Study Participants

This study was conducted between 1 January 2018, and 31 December 2020 within Origyn Fertility Center, Iasi, Romania. A total of two hundred and five women (mean–33.45, range 21–43 years) participated. The AMH level varied from 0.11 to 16.00 ng/dL (mean–2.89).

### 2.2. Inclusion Criteria and Limitations

The main inclusion criteria were: (I) tubal infertility; (II) endometriosis; (III) male-component infertility; and (IV) both regular and irregular menstrual cycles.

Even though the fertility status is dependent on the age of the recipient, there exists situations in which the treatment had no effect. Thus, due to heterogeneity between groups, we were unable to perform any statistical analyses.

### 2.3. Study Design

The ovarian stimulation (OS) protocol with follitropin delta began on day two of the menstrual cycle. According to the established methodology, we harvested the hormonal profile (FSH, LF, P4, and E2), and endovaginal ultrasound was performed on the same day. If the hormonal profile was favorable (FSH < 10 and the ovaries do not present an ovarian cyst), the short antagonist (hCG) stimulation protocol was initiated. All doses were administered according to the algorithm provided by the manufacturer. Each patient received the recommended dose of follitropin delta daily, in the afternoon (between 18:30 and 20:00). On day 6 of stimulation (day 7 of the menstrual cycle) the administration of the antagonist (Orgalutran^®^ 0.25 (ganirelix)/day) started in the morning (between 8:00 and 11:00) after harvesting the hormonal profile. Endovaginal ultrasound was performed to monitor the growth of follicles. On day eight and ten of stimulation, the hormonal profile (ES and P4) was harvested again, together with the endovaginal ultrasound to follow the adequate development of the follicles. When were observed through ultrasound more than 3 follicles over 18 mm, ovulation was triggered by administrating hCG Pregnyl 5000 IU. At 36 h, the ultrasound-guided ovarian puncture took place under deep anal sedation.

### 2.4. Ethical Approval

Each woman signed an informed consent concerning the procedures that will take place prior to enrolment. We obtained ethical approval according to legal provisions for this study from the Ethical Committee at the Origyn Fertility Center (118/565/January/10/2021), the agreement being signed by the Medical Director of the center as well. This manuscript was conducted in accordance with the Helsinki Declaration of human rights and National and European regulations on Biomedical Research in force. Women did not receive any remuneration for their participation since it was voluntary.

### 2.5. Statistical Analysis

Data analysis, editing, sorting, and coding was carried out using Microsoft Excel 2010.

## 3. Results

As presented in Table 1, there was a total of 2532 follicles over 18 mm obtained following the administration of follitropin delta in 205 women. From this, 1719 (67.89%) have been attributed to women under 35 years, and 814 (32.14%) in women above 35 years. Continuing with this concept, we centralized a total of 1891 oocytes in both groups–1279 (67.63%) in women that were under 35 years of age and 612 (32.36%) in women above 35 years. Finally, 978 embryos resulted from 205 women, from which 677 (69.22%) correspond to females under 35 years and 301 (30.77%) to females above 35 years, respectively. However, there were several cases in which the protocol was canceled, since either the recipient manifested no response or due to moderate ovarian hyperstimulation syndrome (OHSS); one woman of 36, and 37 years and one of 43 years. Additionally, we performed a series of interventions such as preimplantation genetic testing (PGT) (*n =* 4–1.95%), embryo transfer on day 3 (*n =* 4–1.95%), and freezing all embryos on day 4 (*n =* 1–0.48%) throughout the entire established interval.

**Figure 1:**
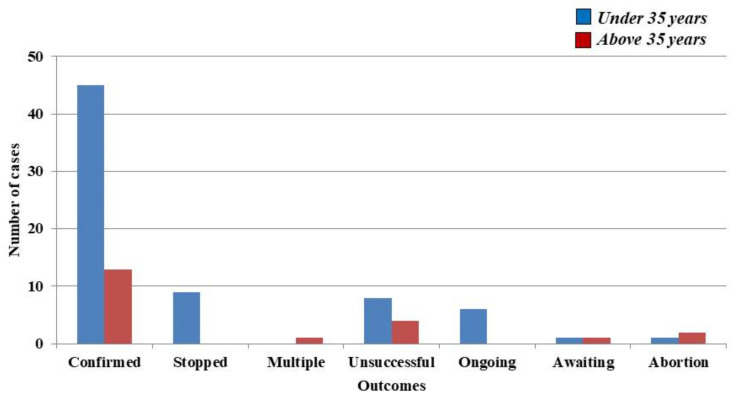
Main outcomes over a period of two years in women that received follitropin delta.

A total of 58 pregnancies were subsequently confirmed, more precisely 45 (36.88%) in women under 35 years and 13 (15.66%) in women above 35 years. Unfortunately, nine pregnancies (7.37%) from the group of women that were under 35 years of ceased to evolve, multiple pregnancies being however noted in one case of a 39-year-old woman with twins (1.20%). Moreover, twelve pregnancies (*n =* 8–6.55%) in females under 35 years of age, and *n =* 4–4.81%) in women above 35 years of age could not be carried out. Six ongoing pregnancies were registered at the time of writing (4.91%) in women under 35 years of age. In both groups there was a case where the result was expected, without any information regarding the status of the current pregnancy (*n =* 1–0.81%, and 1.20%–*n =* 1), respectively. Furthermore, there were also three situations in which mothers suffered an abortion (*n =* 1–0.81%) in women under 35 years of age, while (*n =* 2–2.40%) occurred within the group in which women were above 35 years of age. (Figure 1).

## 4. Discussion

As has been presented throughout this manuscript, follitropin delta can be successfully used in OS to achieve a pregnancy in both groups since we achieve 45–(36.88%) in women that were under 35 years of age and 13 (15.66%) in females above 35 years of age. We encountered only three cases when the procedure needed to be stopped (*n =* 2 were non-responders and *n =* 1 exhibited moderate adverse reactions). Even though the number of volunteers is not comparable with that of Martin and co-authors [12], the number of follicles, oocytes, and embryos was higher in women under 35 years compared with those over 35 years even though the groups were unequal; *n =* 1719–95% CI 1.46, *n =* 1279–95% CI 1.17, and *n =* 677–95% CI 0.92 and *n =* 814–95% CI 1.54, *n =* 612–95% CI 1.22, and *n =* 301–95% CI 0.83.

By putting in balance the effectiveness of follitropin alfa and delta, from 824 ongoing pregnancies, approximately 5.6% (*n =* 25) ended electively because of major congenital malformations.

The fact that fifteen women (8.15%) did not respond to treatment at different stages, eight did not achieve a pregnancy (4.81%), and three had abortions (1.80%) suggests that the treatment’s kinetics are modulated by age and weight [13,14,15]. Based on the existing literature, only two more studies we identified in which women with similar conditions were enrolled.

By referring to our results, nine (4.39%) were stopped, twelve pregnancies (5.85%) could not be continued and three (1.46%) had been electively terminated. Thankfully, at the time of writing, a twin pregnancy had been confirmed (0.48%). We also noted six ongoing pregnancies (2.92%), and two (0.97) of we did not know the exact result.

To date, the Evidence-based Stimulation Trial with Human recombinant Follicle Stimulating Hormone (rFSH)in Europe and Rest of World (ESTHER-1) remains the largest research project dedicated to deepening the understanding of follitropin delta’s reliability. The aim was to compare both conventional and standardized methodologies based on AMH level and BMI index using follitropin delta of 150 IU/day. Ongoing pregnancies and live birth rates were similar in both groups with fewer poor responders, respectively, and hyper-responders following the administration follitropin delta via an individualized manner [16].

By using a subset from ESTHER-1, patients had the possibility to undergo new stimulation cycles in ESTHER-2. It has been demonstrated that the treatment-induced incidence rate of anti-FSH antibodies was between 0.8% and 1.1% in cycle 2 and 3. Similar figures were noted after the first cycle as well. This further support the high efficacy of follitropin delta [17].

Using as reference the serum clearance, its agonistic potential was influenced in part by the hepatic asialoglycoprotein receptor (ASGPR). On the other hand, follitropin alfa was unaffected by ASGPR in experimental models [18,19].

Arce et al. [19] conducted last year a study that aimed to establish the daily follitropin delta dose by further providing a similar ovarian response to 150 IU/day follitropin alfa. For this, they analyzed the ovarian response in 1591 IVF/intracytoplasmic sperm injection (ICSI) that underwent OS. It has been demonstrated that daily doses of follitropin delta (10.0 µg (95% confidence interval [CI] 7.9–12.8) and 10.3 µg (95% CI 9.7–10.8)) promoted the formation of an identical number of oocytes as 150 IU/day follitropin alfa in both phases. When they analyzed patients with either normal or high ovarian reserve and no dose changes, the number of oocytes obtained was the same in all three situations: 150 IU/day follitropin alfa and daily doses of follitropin delta of 9.7 µg (95% CI 7.5–12.4) and 9.3 µg (95% CI 8.6–10.1). Between 9.5 and 10.4 µg was considered the optimal dose that corresponds to 150 IU/day follitropin alfa for serum E2 concentration and the number of follicles ≥ 12 mm.

A multinational clinical trial entitled “Follitropin Delta in Long GnRH Agonist and GnRH Antagonist Protocols” (BEYOND) (ClinicalTrials.gov Identifier: NCT03809429) is currently underway in the recruitment phase. Seventeen institutions from seven states are involved in this project and it is scheduled for completion at the end of September this year. In this interventional study were included 415 participants that were randomized, the main objective being to compare the efficacy and safety of follitropin delta and its personalized dosing algorithm in controlled OS for IVF/ICSI by using a long Gonadotropin-Releasing Hormone (GnRH) agonist protocol compared with a short GnRH antagonist protocol.

The emergence of this novel biomolecule used for OS, which improves both capacity to initiate and sustainability towards multiple follicles, is reflected by a decreased number of CS injections and to the detriment of another drug usage [1]. Thanks to the algorithm provided by the producer, we were able to select the optimal dose for each patient.

## 5. Conclusions

In conclusion, follitropin delta proved to be a safety biomolecule that can be used in OS protocols, which is why we encourage the usage on an even larger scale. To the best of our knowledge, this is the first observational study conducted in Romania, but also in the northeastern region of Europe that addresses such a perspective and brings conclusive evidences in this context.

## Figures and Tables

**Table 1:** Ovarian response in women following the administration of follitropin delta.

Parameters	Under 35 Years	Above 35 Years
	(*n =* 122)	(*n =* 83)
AMH level	Mean—3.46	Mean—2.04
	SE—0.22	SE—0.17
	SD—2.53	SD—1.58
	CI 95%—0.45	CI 95%—0.34
	STDev—2.54	STDev—1.59
	SE Mean—0.23	SE Mean—0.17
Duration of stimulation (days)	Mean—10.11	Mean—9.62
	SE—0.17	SE—0.14
	SD—1.91	SD—1.35
	CI 95%—0.34	CI 95%—0.29
	STDev—1.91	STDev—1.36
	SE Mean—0.17	SE Mean—0.15
Daily dose (ng/mL)	Mean—9.18	Mean—10.63
	SE—0.20	SE—0.20
	SD—2.31	SE—1.89
	CI 95%—0.41	CI 95%—0.41
	STDev—2.32	STDev—1.89
	SE Mean—0.21	SE Mean—0.21
Follicles >18 mm	*n =* 1719	*n =* 814
	Mean—14.09	Mean—9.92
	SE—0.73	SE—0.77
	SD—8.15	SD—7.00
	CI 95%—1.46	CI 95%—1.54
	STDev—8.15	STDev—7.01
	SE Mean—0.74	SE Mean—0.77
Oocytes retrieved	*n =* 1279	*n =* 612
	Mean—10.48	Mean—7.65
	SE—0.59	SE—0.61
	SD—6.57	SD—5.50
	CI 95%—1.17	CI 95%—1.22
	STDev—6.58	STDev—5.51
	SE Mean—0.60	SE Mean—0.62
Embryo day 3	*n =* 677	*n =* 301
	Mean—5.73	Mean—4.01
	SE—0.46	SE—0.41
	SD—5.08	SD—3.62
	CI 95%—0.92	CI 95%—0.83
	STDev—5.09	STDev—3.63
	SE Mean—0.47	SE Mean—0.42

AMH = The Anti-Müllerian Hormone.

## Data Availability

The datasets used and analyzed during the current study are available from the corresponding author on reasonable request.
